# Transactions of the American Dental Association

**Published:** 1893-06

**Authors:** 


### Transactions of the American Dental Association.

The volume of Proceedings of this Association has just come to hand, and is in very good form indeed. It, as well as its predecessors, makes a valuable addition to these volumes from year to year, and not only this but the account of the proceedings pertaining to various questions is always of interest to every progressive dentist. This work from year to year passes into the hands of the members, but it should, far more than it does, be in possession of many in the profession who are not members of this body that they may the better know and understand what is going on in various lines of thought and action in this the representative body of dental science and art in this Country.

The volume can be obtained by application to the Secretary, Dr. Geo. H. Cushing, of Chicago.

